# A Novel Hydroxamic Acid-Based Curcumin Derivative as Potent Histone Deacetylase Inhibitor for the Treatment of Glioblastoma

**DOI:** 10.3389/fonc.2021.756817

**Published:** 2021-11-05

**Authors:** Hao Wang, Lei Shi, Zhimin Wang

**Affiliations:** ^1^The Department of Neurosurgery, The Second Affiliated Hospital of Bengbu Medical College, Bengbu, China; ^2^Department of Neurosurgery, Affiliated First People’s Hospital of Kunshan, Gusu College of Nanjing Medical University, Suzhou, China; ^3^Department of Neurosurgery, Dushu Lake Hospital Affiliated to Soochow University, Suzhou, China

**Keywords:** derivative, glioblastoma, histone deacetylase inhibitor, curcumin, hydroxamic acid

## Abstract

Glioblastoma (GBM) is one of the most common primary and deadliest malignant brain tumor with chemoresistance and poor prognosis. There is a lack of effective chemotherapeutic drug for the treatment of GBM. In this work, we reported the preparation of a histone deacetylase (HDAC) inhibitor, DMC-HA, from the structural modification of natural product curcumin. DMC-HAs were tested in an HDAC inhibition assay and an 3-(4,5-dimethylthiazol-2-yl)-2,5-diphenyltetrazolium bromide (MTT) assay for cytotoxicity. It showed potent inhibition of HDAC1–2 and HDAC6 with IC_50_ values in the submicromolar concentration range. DMC-HA significantly inhibited the proliferation of human glioblastoma U87 cells and mediated apoptosis of U87 cells in a dose- and time-dependent manner. In addition, DMC-HA elevated the acetylation level of histone H3 in U87 cells. Pharmacokinetic studies showed that DMC-HA possessed acceptable pharmacokinetic profiles, accompanied with certain brain permeability. Lastly, we showed that DMC-HA suppressed the growth of tumor in U87 tumor xenograft model *in vivo* with no obvious toxicity. These results demonstrate that DMC-HA has the potential to be developed as a chemotherapeutic drug for GBM patients.

## Introduction

Glioblastoma (GBM) is one of the most common primary and deadliest malignant brain tumor with striking genomic instability and therapeutic resistance. It remains a malignancy with poor prognosis despite that great progress has been made with chemotherapy, radiotherapy (RT), and surgical interventions (1). Although a survival benefit from temozolomide (TMZ) plus RT has been observed in patients whose tumors contained a methylated DNA repair enzyme (2), many patients succumb to this disease after a combination treatment of RT and TMZ with a median survival of only approximately 15 months, and the survivors experience severe side effects from the current treatment options (3). GBM tumor cells show inherent heterogeneous, highly invasive, and resistance to the current regimens (4). A larger number of clinical trials have been performed to investigate the efficacy of novel therapies, but only limited success has been achieved with prolonged survival in GBM patients. Thus, there are great urgent needs to develop novel therapeutic approaches to improve the quality of life of GBM patients (5).

Epigenetic mechanisms, such as acetylation and deacetylation of histones, play an essential role in the epigenetic regulation of gene expression. Histone deacetylases (HDACs) and histone acetyltransferases (HATs) determine the acetylation status of histones, regulating the transcription activation (6). HDACs are a group of zinc-binding metalloenzymes that catalyze the removal of acetyl groups from histones, resulting in chromatin compaction and transcriptional repression of various genes, including those implicated in the regulation of cell survival, proliferation, cell cycle arrest, and apoptosis (7). HDACs are overexpressed in several cancer types (8), and HDAC inhibitors can selectively alter gene transcription in part by chromatin remodeling, thus changing the structure of transcription factor complexes and resulting in cell growth arrest, reduced migration/invasion, angiogenesis, induction of apoptosis, and inhibition of DNA repair (9). To date, five HDAC inhibitors have been used in the clinical practice. Vorinostat, belinostat, romidepsin, and chidamide are approved for the treatment of cutaneous or peripheral T-cell lymphoma and panobinostat for multiple myeloma (10). Moreover, there are growing clinical trials underway to investigate HDAC inhibitors as single agents or in combination with other drugs to treat non-hematological tumors (11).

HDAC inhibitors are emerging as a promising strategy for the treatment of GBM (12). In the past several years, various studies have exhibited the rational and robust targeted therapy of HDAC inhibitors in the treatment of GBM (13). Mutations in histone H3 have been proven by Capdevielle et al. to be oncogenic drivers in diffuse midline glioma, and targeting this epigenetic abnormality by HDAC inhibitors is a potential therapeutic regimen for diffuse midline glioma treatment through regulating scaffolding proteins EBP50 and IRsp53 (14). A combined therapy approach involving HDAC inhibitors and bromodomain protein (BRD) inhibitors has been identified based on a transcriptome and subsequent gene set enrichment analysis in patient-derived xenograft and stem-like glioblastoma cells. This combination treatment reduces tumor growth in orthotopic patient-derived xenograft of GBM and warrants further clinical trials (15). Preclinical evidence shows that HDAC inhibitor vorinostat has antitumor activity against malignant glioma cell lines *in vitro* and orthotopic glioma xenograft *in vivo* (16). The clinical study also showed that vorinostat is well tolerated as a monotherapy in patients with recurrent GBM and exhibited modest single-agent activity (17). In addition, a phase I/II clinical trial showed that suberoylanilide hydroxamic acid (SAHA, vorinostat) combined with standard chemoradiation had acceptable tolerability in newly diagnosed glioblastoma (18).

Although five HDAC inhibitors have been approved and more than 20 new HDAC inhibitors are currently under preclinical and clinical investigations against various cancer, none of these HDAC inhibitors are specifically developed for the treatment of GBM; therefore, the development of new HDAC inhibitors for GBM is an ongoing opportunity and challenge.

Natural products are a great treasure for the discovery of anticancer drugs (19). Indeed, several natural products have been found to exhibit anticancer activities by affecting HDACs, like resveratrol, heliomycin chalcones, and curcumin (20). Curcumin is a polyphenol extracted from turmeric, which exhibits diverse and broad pharmacological activities (21). A few studies have identified HDAC as one of the targets of curcumin, and it downregulates the expression of HDAC (22). Despite its poor bioavailability, curcumin has been identified to suppress medulloblastoma growth *in vivo* through its HDAC inhibition activity (23). In addition, curcumin has been proven to inhibit cell proliferation, invasion, angiogenesis, and metastasis of GBM in our previous work; thus, we hypothesis that curcumin could be developed as an HDAC inhibitor for the treatment of GBM (24–26). However, the poor HDAC inhibition activity and selectivity impeded the direct application of curcumin as an HDAC inhibitor for the treatment of GBM. Furthermore, another major drawback of curcumin is its extremely low oral bioavailability, translating to limited efficacy *in vivo*.

**Figure 1:**
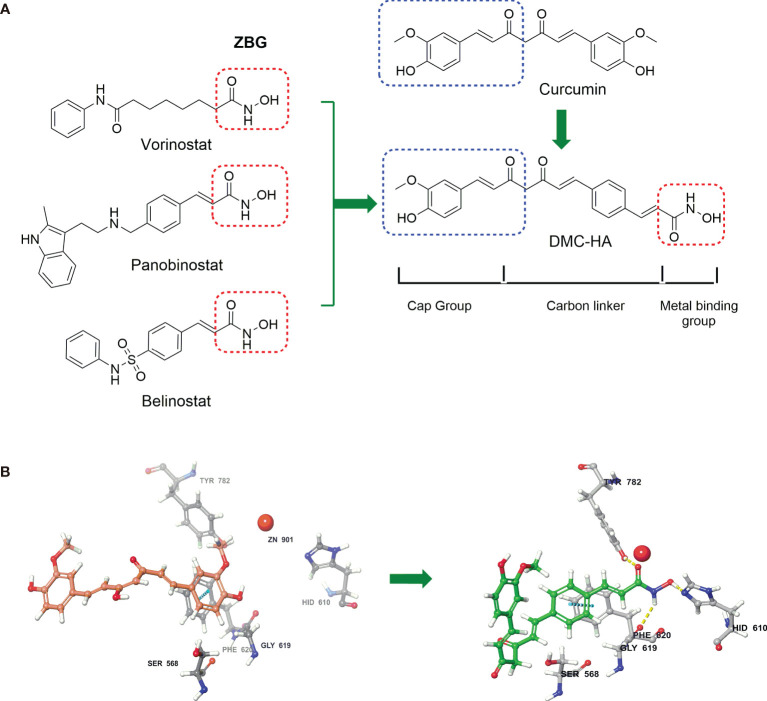
Curcumin-based HDAC inhibitor, DMC-HA, was designed from three FDA approved HDAC inhibitors. **(A)** Pharmacophore model for HDAC inhibitors was applied to the structure of DMC-HA. **(B)** Proposed binding model of curcumin (violet) and DMC-HA (green) with HDAC (PDB code: 5edu).

Based on these observations, we speculate that structural modification of curcumin to be a potent HDAC inhibitor with improved druggability is a feasible approach to apply it to the treatment of GBM. Given the fact that hydroxamic acid is an essential pharmacophore for HDAC inhibition, a novel hydroxamic acid-based curcumin derivative, DMC-HA (**Figure 1**), was designed and synthesized. Herein, we would like to report the discovery and biological evaluation of DMC-HA as a potent HDAC inhibitor for the treatment of GBM.

## Materials and Methods

### Cell Lines and Culturing Conditions

Human GBM U87 cell line was purchased from the American Type Culture Collection (ATCC). Cells were cultured in Dulbecco’s modified Eagle’s medium (DMEM) supplemented with 10% fetal bovine serum (FBS) and maintained in a 37°C, 5% CO_2_ incubator. After cells were routinely passaged at 2- to 3-day intervals, experiments were carried out when the cells entered exponential growth phase.

### HDACs Inhibition Assay

The inhibition assays for HDACs were carried out according to standard protocols. Briefly, purified HDACs were incubated with the test compounds and a carboxyfluorescein-labeled peptide (1 μM) as substrate for 18 h at 25°C in a buffer consisting of 100 mM HEPES (pH 7.5), 1 mg/ml bovine serum albumin (BSA), 0.01% Triton X-100, 1% dimethyl sulfoxide (DMSO), and 25 mM KCl. The reaction was stopped by the addition of 45 μl of 100 mM HEPES (pH 7.5) and 0.08% sodium dodecyl sulfate (SDS). The substrate and the product were then separated electrophoretically using a LabChip 3000 system (Caliper Life Sciences, Hopkinton, MA) with blue laser excitation and green fluorescence detection.

### Cell Viability Assay

Cells (5 × 10^4^) were seeded into 96-well plates and exposed to vehicle (PBS) or different concentrations of compounds for 72 h. Cell viability was measured using the 3-(4,5-dimethylthiazol-2-yl)-2,5-diphenyltetrazolium bromide (MTT) assay and was expressed as a ratio to the absorbance value at 570 nm of the control cells.

### Cell Apoptosis Assay by Flow Cytometry

For apoptosis assays, 5 × 10^5^ U87 cells were seeded into six-well plates for 24 h and treated with a range of concentrations of DMC-HA for 36 h. Annexin V-FITC/PI apoptosis detection kit was used to perform the apoptotic assay following the manufacturer’s instructions (Beyotime, Jiangsu, China). After treatment, 1 × 10^6^ cells were washed using 1 ml binding buffer for three times, then centrifuged at 300×*g* and stained with 10 μl Annexin V-FITC solution at 37°C for 15 min. Before detecting, 5 μl PI solution was added to the samples, and the apoptotic cells were detected using flow cytometry (FACSCalibur BD, BD Biosciences, San Jose, CA, USA).

### Cell Cycle Analysis

For cell cycle analysis, U87 cells (5 × 10^5^ cells/well) were seeded into six-well plates and treated with the indicated concentrations of DMC-HA for 24 h. Cells were detached, fixed in 70% ethanol in PBS (−20°C) overnight and resuspended in PBS supplemented with 100 µg/ml RNase and 50 µg/ml PI for 30 min. Cell cycle analysis was performed on a flow cytometry (FACSCalibur BD, BD Biosciences, San Jose, CA, USA).

### Hepatic Microsome Stability Assay

Metabolic stability was assessed in the presence of human, mouse, and rat liver microsomes (XenoTech, Lenexa, KS, USA). All liquid dispenses and transfer steps were performed with the Freedom Evo automated liquid handler (Tecan US). Compound stock solutions were initially prepared in 100% DMSO and subsequently diluted in acetonitrile for the assay. The pH of the reactions was kept at 7.4 with potassium phosphate buffer. The reaction wells were prepared by adding microsomes to a well and allowed to warm to 37°C. Then, compound was added to each well. The reactions were stirred by adding cofactor reduced nicotinamide adenine dinucleotide phosphate (NADPH) to the reaction well containing microsomes and compounds. Negative controls received buffer only. Immediately after reactions started, 0-min aliquots were promptly collected and mixed in a separate well with ice cold acetonitrile to quench the reactions. The remainder of the reaction volume was incubated at 37°C with shaking. An additional aliquot was collected at 60 min after the start of the reaction and promptly quenched with ice cold acetonitrile. Samples were vortexed and centrifuged at 3,500 rpm for 10 min. The amount of compound in the supernatant was determined by liquid chromatography–tandem mass spectrometry (LC/MS/MS) (Thermo Fischer Scientific, San Jose, CA, USA), and the percent of parent compound remaining after 60 min was calculated. Results are the mean of each reaction triplicate, normalized to the internal standard, and expressed as the percent of compound remaining after the incubation time.

### Pharmacokinetic Study

The animal studies were approved by the Institutional Animal Care and Used Committee. The pharmacokinetic study of DMC-HA was carried out with female SD mice. The compound (2.0 mM) was suspended in DMSO (10%) and Tween 80 (10%) in PBS (80%). A single dose of 1 mg/kg was administrated by intravenous injection (n = 3 mice), followed by blood collection *via* retro-orbital bleeding at 0.25, 0.5, 2.0, 4.0, 8.0, 12.0, and 24 h. Whole blood samples were added to a tube containing ethylenediaminetetraacetic acid (EDTA) and centrifuged. Plasma was collected and stored at −20°C. Compound standards were prepared with pooled plasma samples. Fifty microliters of thawed plasma and standards were added into wells of Ostro Pass-Through Sample Preparation Plate (Waters) for solid phase extraction. Acetonitrile (150 μl) containing internal standard was added and mixed by pipette. Next, clear sample solution was eluted by positive pressure processor under 60 psi for 5 min. The sample solution can be used directly for LC-MS/MS analysis.

### Western Blot Analysis for H3-Histone Acetylation

U87 cells (5 × 10^5^ cells/ml) in six-well plates were incubated with DMC-HA and lysed in radioimmunoprecipitation assay (RIPA) buffer containing protease and phosphatase inhibitors. Protein contents in the lysates were determined by Bradford’s assay, and 50 μg was separated by SDS polyacrylamide gel electrophoresis (SDS-PAGE) electrophoresis. Proteins were transferred to membranes and probed with human anti-acetylated H3 and anti-β actin antibodies as a loading control. Membranes were then labeled with the appropriate horseradish peroxidase (HRP)-conjugated immunoglobulin G (IgG), and protein bands were visualized using enhanced chemiluminescence. (Bio-Rad, Hercules, CA, USA).

### *In Vivo* Study

Six-week-old BALB/c nude mice were purchased from Shanghai Experimental Animal Center of Chinese Academy of Science (Shanghai, China). All animal protocols were implemented according to the guidelines of the Association for Assessment and Accreditation of Laboratory Animal Care. U87 cells (3 × 10^6^) were subcutaneously implanted into the right flank of mice. After 6 days of tumor growth, tumors grew to approximately 100 mm^3^, and mice were randomly divided into three groups with nine animals in each group. Mice in two groups were intraperitoneally injected with 20 mg/kg vorinostat or DMC-HA, respectively, every day for a total of 13 days. The control group was intraperitoneally injected an equal volume of DMSO. Body weights and tumor volumes were measured every 2 days. After treatment, all of the mice were sacrificed, and the tumors were harvested, weighted, and photographed. The tumor volume was calculated according the formula: tumor volume = (length × width^2^)/2.

### Statistical Analysis

All tests were performed using SPSS Graduate Pack 11.0 statistical software (SPSS, Chicago, IL, USA). Descriptive statistics, including the mean and SE, in addition to one-way ANOVAs, were used to determine significant differences. *p < 0.05 and **p < 0.01 were considered statistically significant.

## Results

### Design and Synthesis of Curcumin-Based HDAC Inhibitor

We started our project with reference to the well-established cap-linker-metal binding group pharmacophore model for HDAC inhibitors. This model represented by a capping group is able to interact with the rim of the catalytic tunnel of the enzyme, opposite to a zinc-binding group (ZBG) at the bottom of the catalytic cavity, and a carbon linker connecting the two parts (27). To date, numerous studies have established that hydroxamic acid is a powerful Zn-chelating group; three Food and Drug Administration (FDA)-approved HDAC inhibitors (vorinostat, panobinostat, and belinostat; **Figure 1A**) also contain this ZBG (28). The predicted docking pose of curcumin (**Figure 1B**) suggested that one of the phenyl of curcumin was positioned next to the zinc cation; thus, we envisaged that insertion of hydroxamic acid group into one of the benzene rings of curcumin may facilitate its occupation of the catalytic site and enhance the HDAC binding affinity, leaving the second phenyl group toward the solvent space (capping region). Finally, the structure of curcumin-based HDAC inhibitor is given in **Figure 1**, namely, DMC-HA.

### HDAC Inhibition Assay

To date, 18 HDAC enzymes have been identified and grouped into four classes. Class I (HDACs 1–3 and 8), class II (HDACs 4, 5, 7, and 9 as class IIa and HDACs 6 and 10 as class IIb), and class IV (HDAC 11) are Zn-dependent isozymes, while class III isozymes (Sirt1-7) were quite different from the other ones as they utilize NAD as a cofactor. We systematically investigated the *in vitro* enzymic inhibitory activity of DMC-HA on different class of HDAC isoforms. The inhibitory activity against HDACs 1, 3, 4–6, 8, 10, and 11 subtypes and SIRT1 of DMC-HA was measured, and the results are shown in **Table 1**. Vorinostat is used as a control compound. DMC-HA exhibited IC_50_ values of 0.51, 1.67, 0.38, 2.93, and 8.39 μM against HDACs 1, 3, 6, 8, and 10, respectively, while the IC_50_ values on the four other subtypes exceeded 10 μM. Although both vorinostat and DMC-HA are inert to HDAC4 and HDAC5, it has been reported that class IIa (including HDACs 4, 5, 7, and 9) enzymes are inefficient on histone substrates; class I HDACs, especially HDACs 1–3, and HDAC 6 (class IIb) are considered key targets for cancer treatment (29). Overall, the current biochemical data showed that DMC-HA is a potent inhibitor of class I and class IIb isoforms.

**Table 1:** *In vitro* HDAC enzyme inhibitory activity of DMC-HA.

Compd.	IC_50_ (μM) *^a^*								
	HDAC1	HDAC3	HDAC4	HDAC5	HDAC6	HDAC8	HDAC10	HDAC11	SIRT1
**Vorinostat**	0.19	0.32	>10	>10	0.02	0.34	0.76	0.89	>10
**Curcumin**	23.81	–*^b^*	38.18	–	–	–	>50	28.10	–
**DMC-HA**	0.51	1.67	>10	>10	0.38	2.93	8.39	>10	>10

a Values are the mean of three experiments.

b –, not determined.

### Antiproliferative Efficacy of DMC-HA on Cell Lines

As DMC-HA was identified as a potent novel inhibitor for HDACs, its antiproliferative activities on a panel of cancer cell lines and normal cells were evaluated, including four glioma cell lines, and the results are shown in **Table 2**. Vorinostat and curcumin were also tested as positive controls. Curcumin had moderate antiproliferative activities with IC_50_ values at the range of 16.4–49.2 μM against all eight cell lines, whereas its hydroxamic acid derivative, DMC-HA, exhibited more potent activities with IC_50_ values below 5.3 μM against these cell lines, suggesting a remarkable potency increase. In particular, DMC-HA is a potent compound against four glioma cell lines, with IC_50_ of 2.99 in U251, 2.78 μM in U87, 4.62 μM in SHG44, and 3.38 μM in LN229 cell line, which were comparable with that of positive control vorinostat. More importantly, DMC-HA exhibited low toxicity to human umbilical vein endothelial cells (HUVECs), suggesting the safety of DMC-HA.

**Table 2:** Antiproliferative activity of DMC-HA against several cancer cell lines.

Cell lines	IC_50_ (μM)		
	Vorinostat	Curcumin	DMC-HA
**Human lung cancer cell line A549**	1.13 ± 0.21	32.9 ± 3.99	5.33 ± 0.93
**Human liver cancer cell line HepG2**	2.8 ± 0.43	18.3 ± 3.21	4.28 ± 0.52
**Human leukemia cell line K562**	0.22 ± 0.12	16.4 ± 1.23	1.95 ± 0.71
**Human colon carcinoma cell line SW-620**	0.51 ± 0.03	49.2 ± 7.42	3.22 ± 0.30
**Human glioblastoma cell line U251**	2.62 ± 0.59	27.6 ± 3.11	2.99 ± 0.48
**Human glioblastoma cell line U87**	3.82 ± 0.73	31.9 ± 2.90	2.78 ± 0.81
**Human glioma cell line SHG44**	4.23 ± 0.92	24.6 ± 4.52	4.62 ± 0.72
**Human glioma cell line LN229**	2.90 ± 0.18	21.9 ± 4.65	3.38 ± 0.29
**Human umbilical vein endothelial cells HUVEC**	8.71 ± 0.98	58.5 ± 7.92	27.43 ± 3.94

### Effect of DMC-HA on Histone H3 Acetylation in Cells

**Figure 2:**
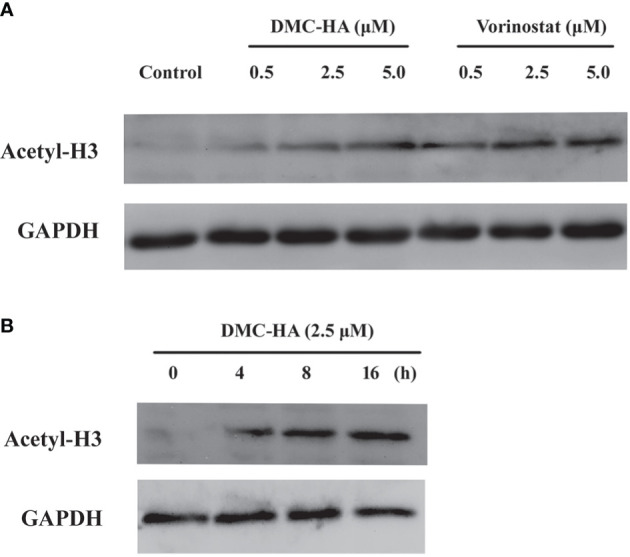
**(A)** Dose-dependent effect of DMC-HA and vorinostat on histone H3 acetylation. **(B)** Time-dependent effect of DMC-HA on histone H3 acetylation at the concentration of 5.0 μM.

Next, DMC-HA-induced acetylation of histone H3 in living cells was analyzed using Western blot method. We compared the inhibition capacities of DMC-HA and vorinostat against HDACs in U87 cells first. After the incubation with various concentrations of DMC-HA or vorinostat, acetylated H3 levels in U87 cells were tested. As shown in **Figure 2A**, DMC-HA and vorinostat dose dependently increased the acetylation of histone H3 after the cells were treated for 24 h. However, it seems that vorinostat more significantly elevated the acetylation of histone H3 compared to that of DMC-HA at the same concentrations. Then, the acetylation of histone H3 after treatment with 5 μM DMC-HA for different times were detected, and the results showed that it increased in a time-dependent manner (**Figure 2B**).

### Cell Cycle Profiles of U87 Cells Treated With DMC-HA

**Figure 3:**
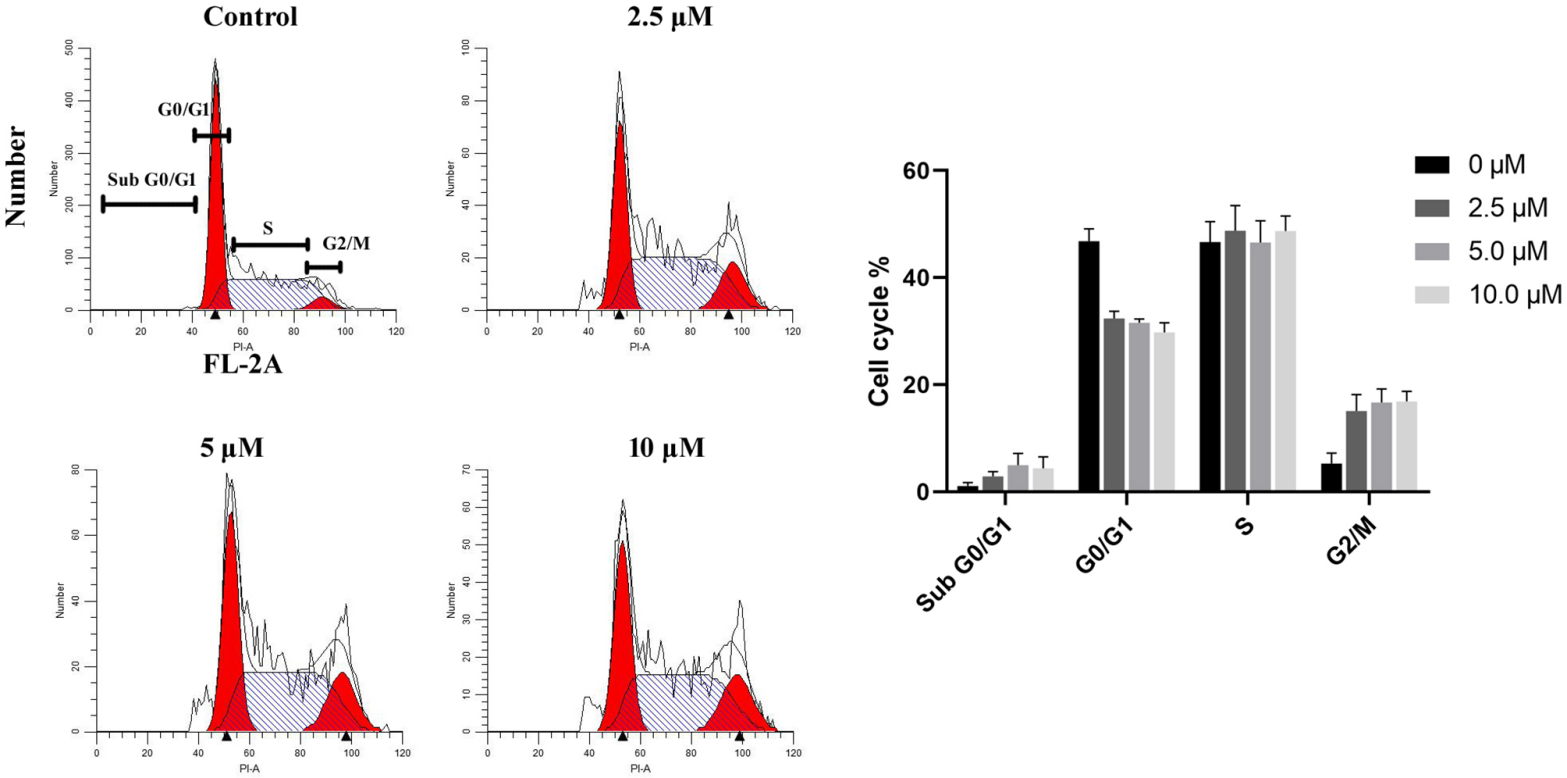
DMC-HA induces G2/M phase arrest. Cell cycle profiles of DMC-HA incubated U87 cells were analyzed by flow cytometry. Cells were exposed to DMC-HA at the indicated concentrations for 24 h (right). Quantitative analysis of cells (left). Data were represented as mean ± SD of three independent experiments.

Inhibition of HDACs results in anticancer effect through various mechanisms, like cell cycle arrest, reduced proliferation, and induction of apoptosis (30). Thus, the cell cycle profile of the human GBM cell line U87 treated with DMC-HA was analyzed by using flow cytometry. As reported in **Figure 3**, after incubation with different concentrations of DMC-HA for 36 h, DNA content analysis of cells revealed a strong increase in G2/M phase population and an associated decrease in G0/G1 population. Although in control cells, only 4.6% of cells were in G2/M phase, 14.5% of cells in G2/M phase were found after treatment with 2.5 μM DMC-HA. Besides, an increase in sub-G0/G1 population, an indication of cell apoptosis, was also observed after the incubation of DMC-HA, suggesting that DMC-HA could promote apoptosis.

### DMC-HA Induces Apoptosis in U87 Cells

**Figure 4:**
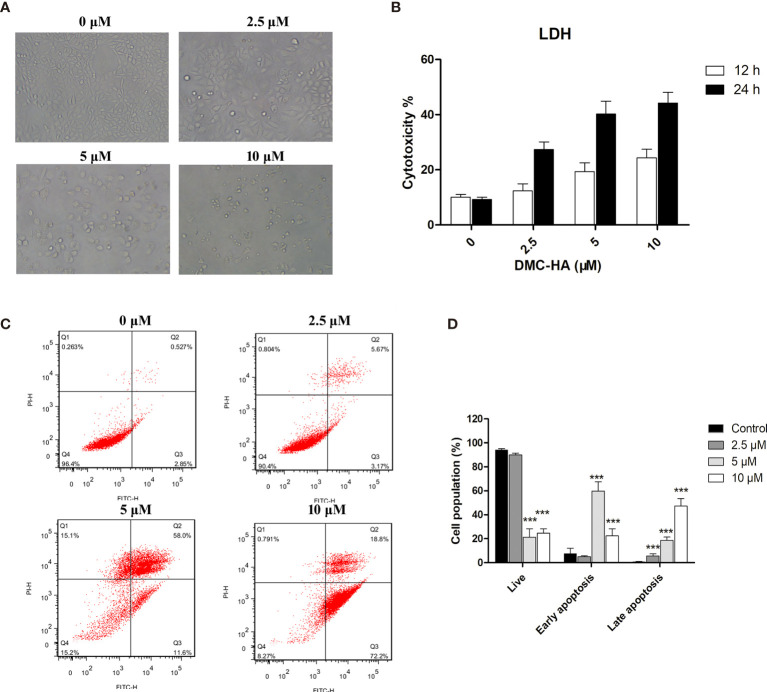
DMC-HA induces apoptosis in U87 cells. **(A)** Phase contrast images of U87 cells incubated with indicated concentrations of DMC-HA for 48 h. **(B)** LDH release as measure of the cytotoxic effect of DMC-HA. **(C)** U87 cells were treated with DMC-HA; after 24 h of treatment, cells were collected and stained with Annexin V/PI, followed by flow cytometric analysis. **(D)** Histograms display the percentage of cell distribution. Data are represented as mean ± SD of three independent experiments. ***p < 0.001 *vs.* control group.

To further investigate the apoptotic effect of DMC-HA on GBM, we treated U87 cells with increasing concentrations of DMC-HA. After 48 h, the morphological changes of DMC-HA-treated U87 cells were observed, such as cell shrinking, rounding, and detachment (**Figure 4A**). In addition, an increase in lactate dehydrogenase (LDH) release was correlated with increasing concentrations of DMC-HA after 48 h of treatment (**Figure 4B**), suggesting that DMC-HA induces cell death in a dose-dependent manner in U87 cells. To further confirm whether DMC-HA could induce apoptosis in U87 cells, cells were stained with Annexin V and PI after treatment with various concentrations of DMC-HA. Flow cytometry analysis of the cells is shown in **Figure 4C**; DMC-HA treatment induced an increase in both early- and late-stage apoptosis of U87 cells, and treatment with 10 μM DMC-HA resulted in an increase in total apoptotic cells from 3.3% up to 90.0% (**Figure 4D**). Together, these results indicated that DMC-HA can trigger apoptosis of U87 cells.

### Microsomal Stability Evaluation of DMC-HA

Structures that are active *in vitro* may not be potent *in vivo* due to their susceptibility to metabolism in the body. We then thoroughly examined the liver microsomal metabolic stability of DMC-HA across three different species (**Table 3**). Curcumin is indeed metabolically labile in mice, rat, and human with short half-life and high clearance rate. However, DMC-HA is relatively more metabolically stable; the half-life in three species is two to five times longer than those of curcumin, suggesting that the introduction of the hydroxamic acid group might block the metabolically labile sites and increase the metabolically stability. In addition, the half-life of DMC-HA in human microsome is generally two to three times longer than those in mice and rats. Collectively, the *in vitro* liver microsomal stability of DMC-HA is favorable, guaranteeing its further *in vivo* studies.

**Table 3:** Microsomal stability of DMC-HA.

Compd.	Human Liver Microsomal Stability		Mice Liver Microsomal Stability		Rat Liver Microsomal Stability	
	T_1/2_ (min)	CL_int_ (μl/min/mg)	T_1/2_ (min)	CL_int_ (μl/min/mg)	T_1/2_ (min)	CL_int_ (μl/min/mg)
**Curcumin**	17.2	82.0	15.3	92.9	10.4	138.2
**DMC-HA**	91.6	17.5	32.7	43.2	40.7	34.2

### Pharmacokinetic and Distribution Study of DMC-HA

As DMC-HA exhibited adequate metabolic stability in microsomal stability assay, we decided to evaluate its *in vivo* pharmacokinetic profiles and assess the relationship between *in vitro* and *in vivo* pharmacokinetic data. The preliminary *in vivo* pharmacokinetic study for DMC-HA was performed in mice. SD mice were intravenously injected with 1 mg/kg DMC-HA or orally administrated with 10 mg/kg DMC-HA. Plasma samples were collected and analyzed with LC-MS/MS. Following p.o. administration, DMC-HA reached its peak concentration of about 1.0 μg/ml near 40 min, and moderate plasma clearance (8.7 L/h/kg) and half-life (4.1 h) were observed (**Table 4**). In addition, DMC-HA displayed acceptable oral bioavailability (40.2%).

**Table 4:** Pharmacokinetic properties of DMC-HA in mice.

Compd.	Dose	T_max_ (h)	C_max_ (ng/ml)	AUC_0–t_ (ng•h/ml)	AUC_0–∞_ (ng•h/ml)	Cl (L/h/kg)	T_1/2_ (h)	F (%)
**DMC-HA**	i.v. 1 mg/kg	–	–	493	502	1.8	3.2	40.2%
**DMC-HA**	p.o. 10 mg/kg	0.67	1,072	1,982	2,013	8.7	4.1	

**Figure 5:**
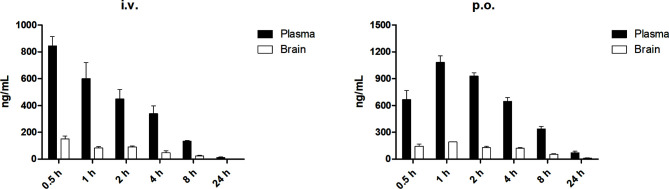
Concentrations of DMC-HA in plasma and brain over time following a single administration in mice. Left, i.v. 5 mg/kg; right, p.o. 10 mg/kg.

Discovery of drugs for the treatment of GBM encounters formidable challenges to their ability to cross the blood–brain barrier (BBB) (31). Thus, we further investigated the distribution of DMC-HA to identify whether it has the ability to cross the BBB. The results in **Figure 5** show that although the brain concentrations of DMC-HA were not high relative to the plasma exposure (brain-to-plasma ratios of 0.08–0.23), it can indeed penetrate the BBB and distribute from plasma into brain tissue. In addition, stable and durable distribution of DMC-HA in the brain was observed after p.o. administration. Nevertheless, optimization for the balance among potency, selectivity, metabolic stability, and brain permeability in a single molecule was challenging. These preliminary results provide valuable information for further structure optimization of DMC-HA and a guidance for its following *in vivo* studies.

### *In Vivo* Antitumor Efficacy of DMC-HA

**Figure 6:**
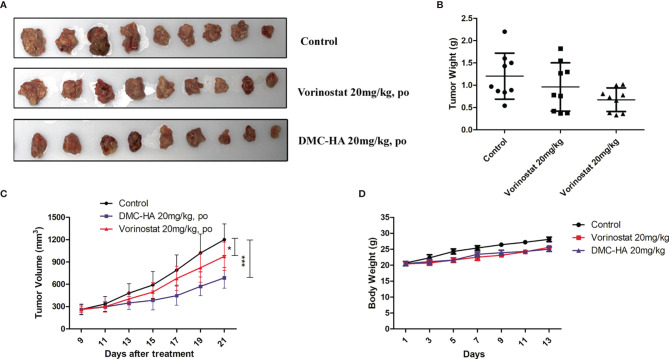
Antitumor activity of DMC-HA. **(A)** Photographs of representative tumors removed from mice in different groups at 21 days after initiation of treatment. **(B)** DMC-HA treatment resulted in significantly lower tumor weight compared with controls. **(C)** Antitumor activity of DMC-HA expressed as tumor volume. **(D)** Body weight changes of mice during treatment. *p < 0.05, ***p < 0.001.

Given the promising *in vitro* properties and good pharmacokinetic profiles of DMC-HA, we evaluated its *in vivo* behavior in U87 tumor xenograft model. U87 cells were subcutaneously inoculated into the right flank of mice. The mice were randomly divided into three groups (*n* = 9); the positive control vorinostat and DMC-HA were administered by the oral route once daily at the dose of 20 mg/kg. The resulting tumors were excised from the animals after treatment (**Figures 6A, B**). Both compounds showed no observable toxicity during the administration period and had no effects on the body weight (**Figure 6D**). Vorinostat at the dose of 20 mg/kg showed moderate inhibition on the tumor volume (24.7%, *p* = 0.04 *vs.* control) (**Figure 6C**). Interestingly, DMC-HA was much more active than vorinostat at the same dose, which inhibited the tumor volume with a value of 52.1% (*p* = 0.00083). Taken together, these data indicated that DMC-HA was efficacious in inhibiting the growth of GBM *in vivo* and deserved further evaluations.

## Discussion

In the present study, we developed a novel HDAC inhibitor, DMC-HA, derived from natural product curcumin. DMC-HA was prepared by introducing a privileged pharmacophore, hydroxamic acid moiety, into the benzene ring of curcumin. We demonstrated that DMC-HA exhibited IC_50_ in the submicromolar to micromolar range against HDACs and micromolar antiproliferative activity against seven cancer cell lines, including four glioma cell lines. The cell cycle profiles of U87 cells showed that DMC-HA induced a strong increment of G2/M population, and it caused a marked increase in apoptosis, which was determined by Annexin V/PI staining. DMC-HA had an acceptable human microsomal stability and oral bioavailability. More importantly, the antitumor efficacy of DMC-HA was demonstrated in U87 xenograft-bearing mice. Our results provide a rational for developing DMC-HA as a new anti-GBM therapy.

GBM is one of the most malignant form of primary brain tumor with extremely poor prognosis. The current treatment guidelines for GBM includes maximal surgical resection followed by radiotherapy and adjuvant chemotherapy. However, due to the lack of effective chemotherapeutic drugs, recurrence is inevitable after standard of care. The median survival of patients with GBM is approximately 8–15 months, and only 3%–5% of patients survive longer than 3 years. This promotes the development of potent chemotherapeutic drugs, which could destroy the residual GBM cells after surgical treatment. A number of targeted anticancer drugs, such as novel small-molecule kinase inhibitors, monoclonal antibodies, FDA-approved drugs, and some natural and synthetic anti-GBM agents are the main focus of researchers nowadays (32).

Analysis of RNA-sequencing data from The Cancer Genome Atlas (TCGA) revealed significant increase in the expression levels of HDACs 1–3 and HDAC 7 in high grade gliomas, implying that HDACs are potential drug targets for GBM therapy (33). Vorinostat was the first inhibitor entering clinical trials to treat GBM; however, the phase II trial of vorinostat with radiotherapy and concomitant TMZ did not meet the efficacy endpoint. In this clinical study, patients received oral vorinostat (300 or 400 mg/day) due to the poor pharmacokinetic profiles of vorinostat, which might be the reason for the failure (18). Panobinostat and valproic acid had also been evaluated in clinical studies using combination strategy but were terminated because of some reasons (34). Nevertheless, many preclinical studies showed significant promise about HDAC inhibitors synergizing with other drugs or alone for GBM treatment (12). Studies found that FK228 could augment TMZ sensitivity *in vivo* and *in vitro* partially by blocking PI3K/AKT/mTOR signal pathway (35). Treatment with tubastatin A, a selective HDAC6 inhibitor, was reported to abrogate TMZ resistance by decreasing and inactivating EGFR protein (36). In addition, Luesch et al. demonstrated that class I selective HDAC inhibitor largazole exhibited *in vitro* antiproliferative activity against GBM cells and sufficient BBB permeability (37). As great challenges and barriers exist in the application of HDAC inhibitors to the treatment of patients with GBM, a better candidate is urgently required. In this study, we designed our HDAC inhibitor based on the well-established cap-linker-metal binding group pharmacophore model for HDAC inhibitors. Hydroxamic acid was selected as the ZBG, which was connected to one of the phenyl of curcumin. We envisaged that insertion of hydroxamic acid group into one of the benzene rings of curcumin may facilitate its occupation of the catalytic site and enhance the HDAC binding affinity. The HDAC enzyme inhibition assay showed that DMC-HA exhibited IC_50_ values of 0.51, 1.67, 0.38, 2.93, and 8.39 μM against HDACs 1, 3, 6, 8, and 10, respectively, suggesting that DMC-HA is a potent pan HDAC inhibitor.

The BBB limits the delivery of systemically administered drugs to the brain, thus excludes the vast majority of the current cancer therapeutics from GBM treatment (38). The poor pharmacodynamic/pharmacokinetic properties of the present FDA-approved HDAC inhibitors makes it challenging to convert them for the treatment of GBM. There is no doubt that drugs with poor BBB permeability could not exert efficacy to the tumor cells within brain. In this study, we investigated the distribution of DMC-HA to identify whether it has the ability to cross the BBB. The results in **Figure 5** show that although the brain concentrations of DMC-HA were not high relative to the plasma exposure, it can indeed penetrate the BBB and distribute from plasma into brain tissue. In addition, stable and durable distribution of DMC-HA in the brain was observed after p.o. administration. These preliminary results provided important information for the further *in vivo* antitumor evaluation of DMC-HA. It is now widely accepted that evaluating whether a drug could across an intact BBB into brain is a critical first step in developing effective therapies for GBM. Pharmacokinetic study must be a key consideration for the newly designed compound as effective therapies for GBM.

Altogether, our study demonstrated that DMC-HA, a novel hydroxamic acid derivative of curcumin, showed potent inhibition of HDACs and suppressed the human glioblastoma cell proliferation. The good bioavailability of DMC-HA and its ability to inhibit tumor growth in a U87 GBM xenograft model implies that DMC-HA warrants further development as an antitumor agent for the treatment of GBM.

## Data Availability Statement

The raw data supporting the conclusions of this article will be made available by the authors, without undue reservation.

## Ethics Statement

The animal studies were approved by the Institutional Animal Care and Used Committee of Soochow University.

## Author Contributions

HW and LS contributed equally to this work. All authors contributed to the article and approved the submitted version.

## Funding

This work was supported by the National Natural Science Foundation of China (81772691, 30200335 and 81370062), China Postdoctoral Science Foundation (2017M620196 and 2018T110467), Key Technologies of Suzhou Science and Technology Development (SS2019050), Kunshan High-Level Medical Talent Post Training Project (2018), Key Young Medical Talents Project in Jiangsu Province (QNRC2016526), and Suzhou Health Personnel Training Project (GSWS2020112). The funders had no role in the study design, data collection and analysis, decision to publish, or in the preparation of the manuscript.

## Conflict of Interest

The authors declare that the research was conducted in the absence of any commercial or financial relationships that could be construed as a potential conflict of interest.

## Publisher’s Note

All claims expressed in this article are solely those of the authors and do not necessarily represent those of their affiliated organizations, or those of the publisher, the editors and the reviewers. Any product that may be evaluated in this article, or claim that may be made by its manufacturer, is not guaranteed or endorsed by the publisher.
